# Absence of Leucine Zipper in the Natural FOXP3Δ2Δ7 Isoform Does Not Affect Dimerization but Abrogates Suppressive Capacity

**DOI:** 10.1371/journal.pone.0006104

**Published:** 2009-07-01

**Authors:** Reiner K. W. Mailer, Kirsten Falk, Olaf Rötzschke

**Affiliations:** 1 Max-Delbrück-Center for Molecular Medicine (MDC), Berlin, Germany; 2 Singapore Immunology Network (SIgN), IMMUNOS, Singapore, Singapore; New York University School of Medicine, United States of America

## Abstract

**Background:**

Phenotype and function of regulatory T cells (Treg) largely depend on the presence of the transcription factor FOXP3. In contrast to mice, human Treg cells express isoforms of this protein. Besides the full length version (FOXP3fl), an isoform lacking the exon 2 (FOXP3Δ2) is co-expressed in comparable amounts. Recently, a third splice variant has been described that in addition to exon 2 also misses exon 7 (FOXP3Δ2Δ7). Exon 7 encodes for a leucine zipper motif commonly used as structural dimerization element. Mutations in exon 7 have been linked to IPEX, a severe autoimmune disease suggested to be caused by impaired dimerization of the FOXP3 protein.

**Principal Findings:**

This study shows that the lack of exon 7 does not affect (homo-) dimerization. Moreover, the interaction of FOXP3Δ2Δ7 to RUNX1, NFAT and NF-kB appeared to be unchanged in co-immunoprecipitation experiments and reporter gene assays, when compared to FOXP3fl and FOXP3Δ2. Nevertheless, retroviral transduction with FOXP3Δ2Δ7 failed to induce the typical Treg-associated phenotype. The expression of FOXP3-induced surface molecules such as CD25 and CTLA-4 were not enhanced in FOXP3Δ2Δ7 transduced CD4+ T cells, which also failed to exhibit any suppressive capacity. Notably, however, co-expression of FOXP3fl with FOXP3Δ2Δ7 resulted in a reduction of CD25 expression by a dominant negative effect.

**Conclusions:**

The leucine zipper of FOXP3 does not mediate dimerization or interaction with NFAT, NF-kB and RUNX1, but is indispensable for the characteristic phenotype and function in Treg cells. FOXP3Δ2Δ7 could play a role in regulating the function of the other FOXP3 isoforms and may be involved in cancer pathogenesis, as it is overexpressed by certain malignant cells.

## Introduction

Peripheral tolerance is maintained by various suppressor cells. One important subset of these cells are regulatory CD4+ T cells (Treg). They differ from ‘conventional’ effector CD4+ T cells in the constitutive expression of the IL-2 receptor α-chain (CD25) and other surface markers such as cytotoxic T lymphocyte-associated antigen 4 (CTLA-4), glucocorticoid-induced TNFR (GITR) and CD39 [1]–[4]. To date the most specific marker is the forkhead box/winged helix transcription factor FOXP3 [5]. FOXP3 largely controls the development into Treg cells. Loss of function due to mutations or complete absence of the transcription factor leads to lethal autoimmune diseases in *scurfy* mice [6] and the often fatal ‘immune dysregulation, polyendocrinopathy, enteropathy, X-linked syndrome’ (IPEX) in humans [7].

Retroviral transduction of CD4+ T cells by FOXP3 is sufficient to induce a Treg phenotype and the capacity to suppress lymphoproliferation [5], [8]. The human protein comprises 431 amino acids (aa). It contains a repressor domain (aa 67–132), a zinc finger (aa 199–222), a leucine zipper motif (aa 239–260) and a conserved DNA binding forkhead domain (FKH) at the C-terminus (aa 335–418). FOXP3 acts as a functional repressor of NFAT- and NF-kB-mediated gene transcription [9] and crystal structure analysis for the homologue FOXP2 suggests that C-terminal DNA-binding is associated with NFAT [10].

In contrast to mice, human Treg cells always display expression of two FOXP3 isoforms. Both are expressed at similar levels of which one representing the full length isoform (FOXP3fl), while the other isoform lacks exon 2 (FOXP3Δ2) [11]. Exon 2 (aa 72–106) associates and represses the function of retinoic acid-related orphan receptors α and γt (RORα, RORγt) [12], [13]. As a consequence the suppression of RORγt-induced Th17 induction is evident only with FOXP3fl [13], [14], most other functions, however, are either redundant or seem to have an additive impact [15]. While both FOXP3fl and FOXP3Δ2 contribute to the phenotype of human Treg cells, the genes affected by the two isoforms seem to overlap only partly.

Recently, a third isoform has been identified lacking in addition to exon 2 also exon 7 [16]. Exon 7 encodes for a leucine zipper motif (aa 239–260; VX_6_LX_6_LX_6_L). The region was thought to be indispensable for FOXP3 function, since the analysis of different IPEX patients unveiled a concentration of fatal FOXP3 mutations in the FKH and the leucine zipper region [17]. Leucine zippers are a common dimerization element [18] and IPEX mutations lacking either a lysine residue at position 250 or a glutamic acid residue at position 251 in fact fail to dimerize during immunoprecipitations [19], [20]. Moreover, the region is also located proximal to another important binding site. The Runt-related transcription factor 1 (RUNX1) is reported to target a region c-terminal to the leucine zipper, which seems to be crucial for the induction of CD25 and the suppression of IL-2 [21].

In this study, we show that, despite the absence of the leucine zipper, FOXP3Δ2Δ7 is still able to dimerize with all FOXP3 isoforms. Moreover, NFAT- and NF-kB-mediated transcription as well as direct binding to RUNX1 are not affected by the absence of exon 7. In contrast to FOXP3fl and FOXP3Δ2, FOXP3Δ2Δ7 fails to induce the expression of CD25 and CTLA-4 and does not mediate suppressive capacity into transduced CD4+CD25− T cells. As FOXP3Δ2Δ7 is able to inhibit the FOXP3fl- or FOXP3Δ2-induced expression in a dominant negative fashion, it may therefore have a regulatory impact on the function of the other two isoforms.

## Results

### Expression and sub-cellular distribution of FOXP3 isoforms

**Figure 1:**
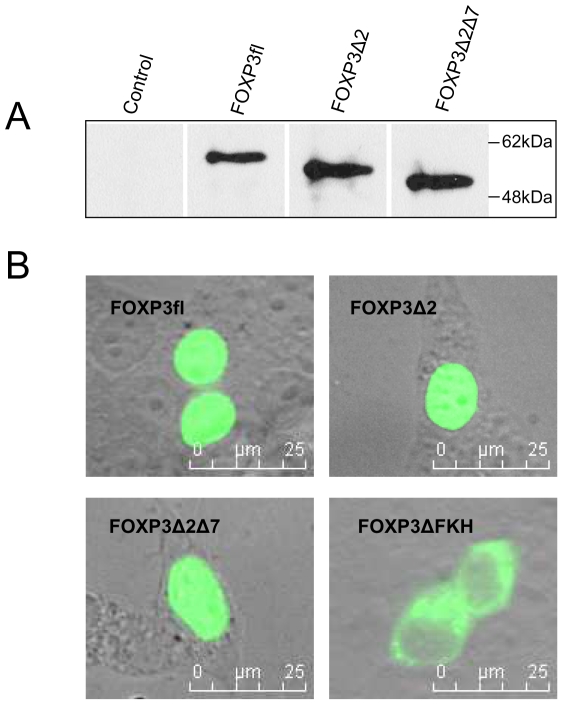
Ektopic expression of FOXP3 isoforms. (A) HEK293T cells were transfected with plasmids encoding for FOXP3fl, FOXP3Δ2 and FOXP3Δ2Δ7 isoforms. Protein levels of splice variants were analyzed by Western blots using isoform-non specific α-FOXP3 antibody eBio7979. FOXP3 protein detection shown is representative for three independent experiments. (B) HEK293T cells were transfected with plasmids encoding for N-terminal GFP fusion proteins of FOXP3fl, FOXP3Δ2, FOXP3Δ2Δ7 and FOXP3ΔFKH. GFP-FOXP3, GFP-FOXP3Δ2 and GFP-FOXP3Δ2Δ7 were translocated into the nucleus, whereas GFP-FOXP3ΔFKH remained in the cytoplasm. Distribution of GFP-FOXP3 fusion proteins shown is representative for three independent experiments.

Human Treg cells express constitutively high levels of the two FOXP3 isoforms FOXP3fl and FOXP3Δ2 [5], [22]. We generated cDNA transcripts of purified Treg cells and identified in addition to these two isoforms a splice variant that lacks exon 2 and also exon 7. The latter was previously described as FOXP3Δ2Δ7 [16]. To test whether all FOXP3 isoforms can be expressed by mammalian cells, HEK293T cells were transfected with pcDNA3.1+ constructs, encoding for FOXP3fl, FOXP3Δ2 or FOXP3Δ2Δ7. Western blot analysis of transfected cells showed that all isoforms can be generated by the cell, migrating during SDS-PAGE with an apparent molecular weight of 58kDa, 54kDa and 50kDa, respectively (Fig. 1*A*). Fusion proteins linked N-terminally to GFP further revealed that all three isoforms are effectively translocated into the nucleus (Fig. 1*B*). Analysis by fluorescence microscopy of HEK293T cells transfected with these constructs showed bright staining of the nucleus, while GFP-FOXP3ΔFKH, a truncated version lacking the DNA binding domain is retained in the cytoplasm [23].

**Figure 2:**
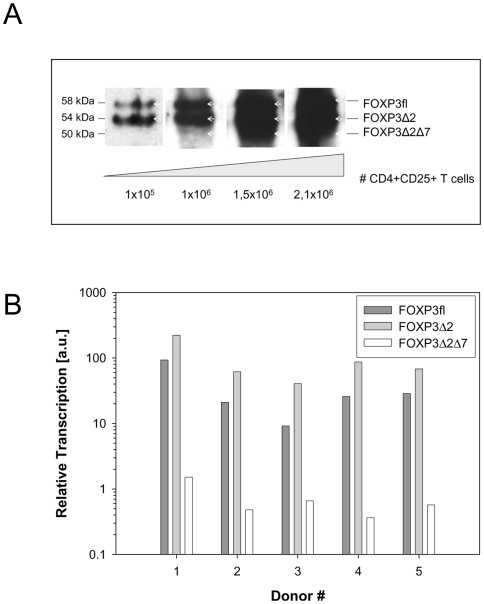
Expression of FOXP3fl, FOXP3Δ2 and FOXP3Δ2Δ7 in human Treg cells. (A) Protein expression levels of FOXP3 isoforms in human Treg cells. CD25+CD4+ T cells were isolated from PBMC by MACS using α-CD25 Treg isolation kit. FOXP3-specific eBio7979 antibody was used after Western blotting. The number of CD25+ cells loaded per lane is indicated. (B) RT-PCR analysis of human CD4+CD25+ cells. Relative transcription levels of FOXP3 isoforms in five healthy donors were investigated.

Western blot analysis of human Treg cells seems to reveal a faint band presumably representing the FOXP3Δ2Δ7 isoform (Fig. 2*A*). However, the experiment did not reveal a clear result regarding the relative amount of FOXP3Δ2Δ7 as at higher lysate concentrations the view was obstructed by the much broader band of FOXP3Δ2 migrating slightly above FOXP3Δ2Δ7. To determine the relative frequency of the three isoforms in human PBMC we therefore performed also an RT-PCR analysis of primary CD4+CD25+ T cells freshly isolated from healthy donors. Primer pairs spanning from exon 1 to exon 2 (FOXP3fl), from exon 1 to exon 3 (FOXP3Δ2) and from exon 6 to exon 8 (FOXP3Δ7) were used to analyse the amount of transcript for each isoform. By comparing 5 different donors a similar pattern for the relative ratios of FOXP3 splice variants was obtained (Fig. 2*B*). In line with previous reports the dominant isoforms were FOXP3fl and FOXP3Δ2. The amounts were comparable although, at least on the transcriptional level, FOXP3Δ2 always exceeded FOXP3fl 2–3 times. In comparison, FOXP3Δ2Δ7 is much less frequent. 15–70 times less transcripts were detected compared to FOXP3fl. In contrast to the isoforms of genes such as CD45 [24] the ratio was apparently unaffected from the activation status of the cells. Stimulation of Treg cells either with α-CD3 antibodies or with PMA/Ionomycin did not change the ratio between isoforms (data not shown).

### Dimerization of FOXP3 is independent of exon 7

**Figure 3:**
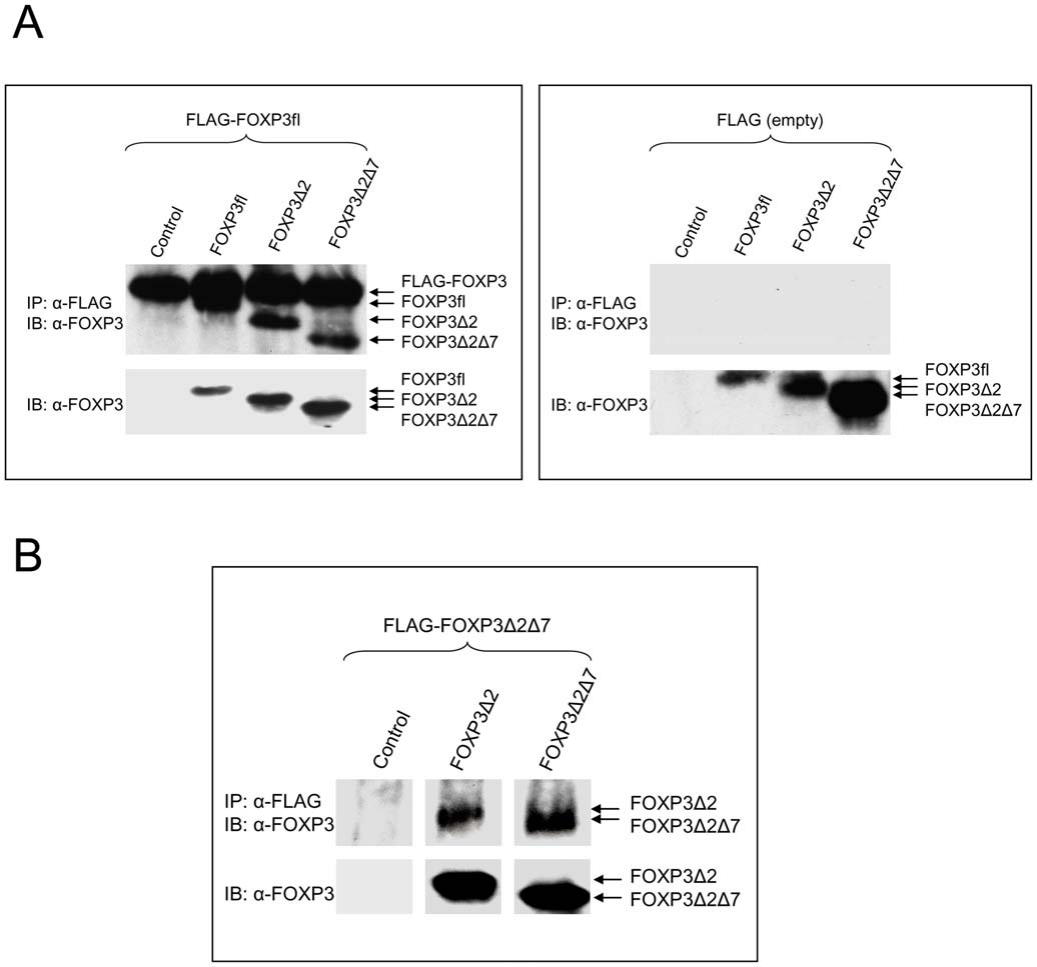
Dimerization of FOXP3fl, FOXP3Δ2 and FOXP3Δ2Δ7 with FOXP3Δ2Δ7. (A) HEK293T cells were co-transfected with FLAG-tagged FOXP3fl (left panel) or empty vector (right panel) and either control vector, FOXP3fl, FOXP3Δ2 or FOXP3Δ2Δ7. Co-immunoprecipitation experiments (IP) were performed with α-FLAG agarose and complexes were analyzed in immunoblots (IB) with FOXP3-specific antibody eBio7979 (upper panels). Expression of non-FLAG-tagged constructs was confirmed by immunoblotting of total cell lysates (lower panels). Co-immunoprecipitation shown is representative for three independent experiments. (B) HEK293T cells were co-transfected with FLAG-tagged FOXP3Δ2Δ7 and either control vector, FOXP3Δ2 or FOXP3Δ2Δ7. IP was performed with α-FLAG agarose and complexes were analyzed in IB as described above. Co-immunoprecipitation shown is representative for three independent experiments.

Since exon 7 encodes for a leucine zipper, it has been suggested that this particular region is necessary for protein-protein-interactions required for homodimerization [19]. To determine the influence of the leucine zipper we, therefore, investigated the ability of the natural isoforms to form complexes with FOXP3Δ2Δ7. FLAG-tagged FOXP3fl was co-transfected with FOXP3fl, FOXP3Δ2 or FOXP3Δ2Δ7 in HEK293T cells, and dimeric FOXP3 complexes were isolated by immunoprecipitation with α-FLAG agarose. Western blot analysis confirmed that FLAG-FOXP3fl binds FOXP3fl, but also revealed that FOXP3Δ2 and, surprisingly, also the leucine zipper deficient FOXP3Δ2Δ7 isoform are able to bind FOXP3fl (Fig. 3*A*). Likewise, in a reversed experiment, also FLAG-tagged FOXP3Δ2Δ7 was able to pull down FOXP3Δ2 and FOXP3Δ2Δ7 (Fig. 3*B*). Similar results were also obtained when the precipitation was carried out with the isoform-specific antibody clone FJK-16s (data not shown). Both exon 2 and exon 7 were apparently not required for dimerization. Thus, at least for homodimerization of FOXP3 the leucine zipper is apparently not required.

### FOXP3Δ2Δ7 represses NFAT- and NF-kB-mediated gene transcription and binds RUNX1

**Figure 4:**
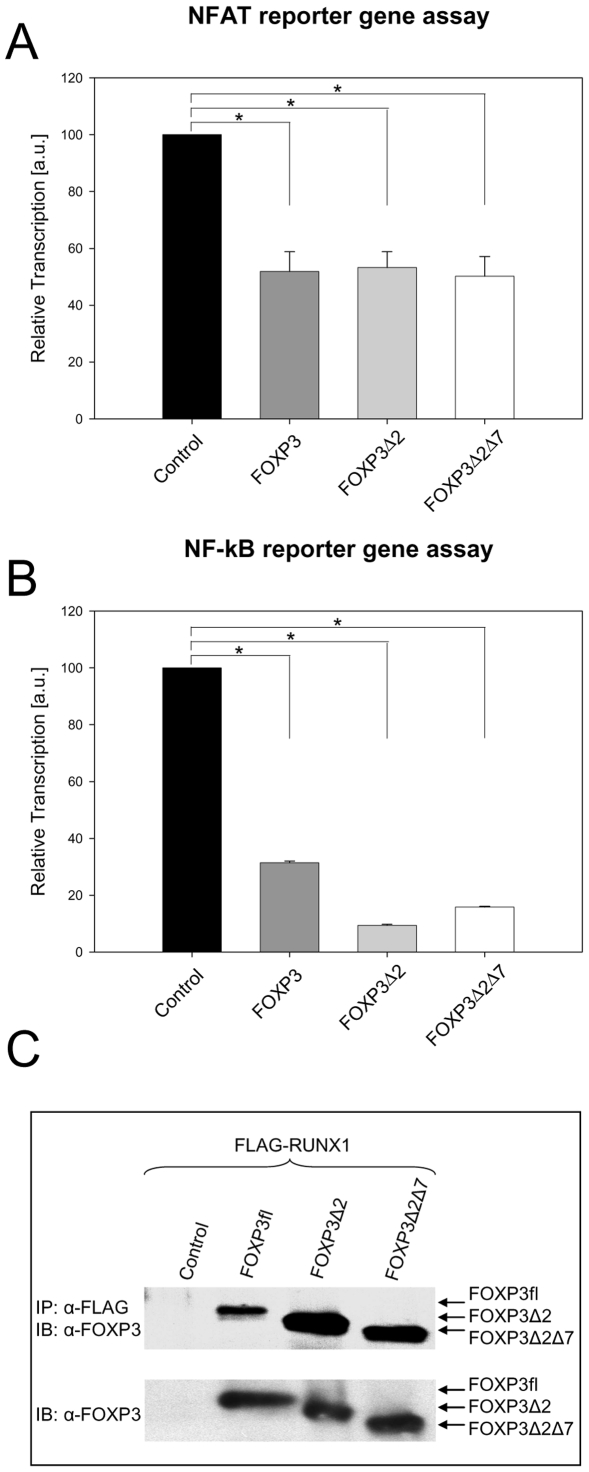
All FOXP3 isoforms interact with NFAT, NF-kB and RUNX1. (A) FOXP3 isoforms repressed NFAT-mediated transcription of luciferase in reporter assays. NIH3T3 cells were co-transfected with either control vector, FOXP3fl, FOXP3Δ2 or FOXP3Δ2Δ7 and NFAT luciferase reporter plasmid. Transcription was normalized to *Renilla* luciferase activity; the average induction in absence of FOXP3 expression was set 100%. Reporter gene assay shown is representative for three independent experiments. Error bars represent SD; statistical analysis was performed with 2-tailed unpaired student's t-test. (B) FOXP3 isoforms repressed NF-kB-mediated transcription of luciferase. Experiments were carried out as above except that a NF-kB luciferase reporter plasmid was used. Reporter gene assay shown is representative for three independent experiments. Error bars represent SD; statistical analysis was performed with 2-tailed unpaired student's t-test. (C) FOXP3 isoforms bind RUNX1 in co-immunoprecipitation experiments. HEK293T cells were co-transfected with FLAG-tagged RUNX1 and either control vector, FOXP3fl, FOXP3Δ2 or FOXP3Δ2Δ7. Co-immunoprecipitation experiments (IP) were performed with α-FLAG agarose and complexes were analyzed in immunoblots (IB) with the FOXP3-specific antibody eBio7979 (upper panels). Expression of non-FLAG-tagged constructs was confirmed by immunoblotting of total cell lysates (lower panels). Co-immunoprecipitation shown is representative for three independent experiments.

For mouse, in vitro studies showed that NFAT- and NF-kB-mediated gene transcription is repressed in the presence of Foxp3. The effect is not observed with the mutant *scurfy* Foxp3 or truncated Foxp3ΔFKH [9], [22]. The reporter gene assays shown here revealed that also the human FOXP3 isoforms are functional repressors (Fig. 4*A, B*). This applied not only for FOXP3fl and FOXP3Δ2 but, most notably, also for FOXP3Δ2Δ7. Independently of the presence of exon 2 and exon 7, all isoforms reduced NFAT-mediated gene transcription to a very similar extent (∼50%), (Fig. 4*A*). In line with this result, also NF-kB-mediated transcription was inhibited (Fig. 4*B*). Co-transfection with FOXP3fl lowered luciferase transcription levels to 31%, while FOXP3Δ2 and FOXP3Δ2Δ7 even decreased the levels to 9 and 16%, respectively.

Another transcription factor known to interact with FOXP3 is RUNX1 [21]. It was recently reported that the physical interaction is apparently indispensable to assemble the active FOXP3-transcription complex [21]. Co-immunoprecipitation experiments revealed that both FOXP3 and FOXP3Δ2 are able to bind the transcription factor. Moreover, although the RUNX1 binding region of FOXP3 is in close proximity to the leucine zipper, also FOXP3Δ2Δ7 was still capable to bind RUNX1 (Fig. 4*C*). Thus, the presence of exon 2 or exon 7 is not required for binding to RUNX1 or the interaction with NFAT or NF-kB.

### FOXP3Δ2Δ7 gene transfer does not confer suppressive capacity

**Figure 5:**
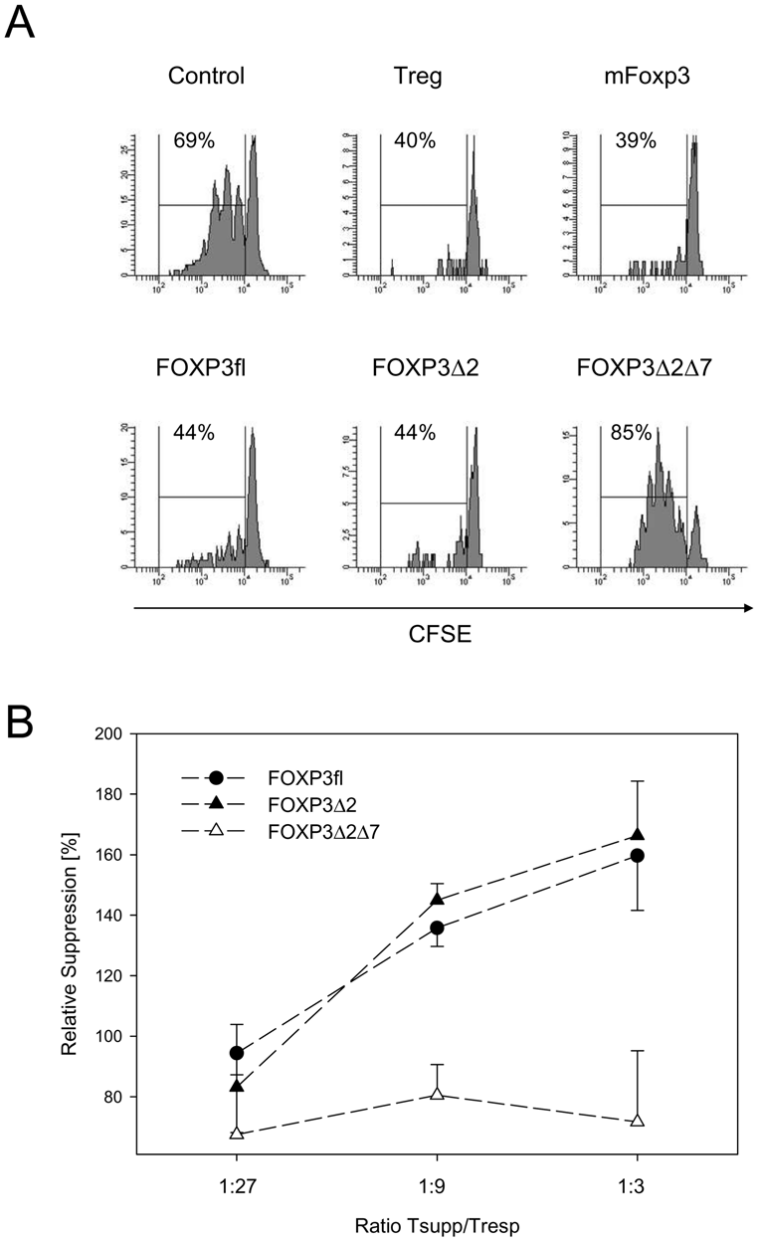
Suppression of CFSE labelled responder cells by FOXP3-transduced T cells. (A) Suppressive capacity of FOXP3 isoforms. Freshly isolated murine CD4+CD25− cells were labelled with carboxy-fluorescein diacetate succinimidylester (CFSE). Lymphoproliferation was detected in co-culture experiments with FACS-sorted FOXP3-transduced CD4+ cells or freshly isolated CD4+CD25+ regulatory mouse T cells in the presence of plate bound α-CD3 and irradiated APC. Ratio of suppressor cells to responder cells was 1∶3. CFSE dilutions were analysed by flow cytometry after 4 days, gating on live CD4+ T cells. Proliferation was suppressed by freshly isolated CD4+CD25+ regulatory T cells (Treg) and CD4+CD25− T cells transduced with murine Foxp3 (mFoxp3), with human FOXP3fl or FOXP3Δ2 but not with T cells transduced with empty control vector (Control) or with FOXP3Δ2Δ7. Suppression assay shown is representative for three independent experiments. (B) Titration of FOXP3-transduced suppressor T cells (Tsupp) and CFSE labelled responder T cells (Tresp). Experiment was carried out as described above except that T cells transduced with FOXP3fl, FOXP3Δ2 or FOXP3Δ2Δ7 were titrated to indicated ratios. The experiment was carried out in triplicates; error bars represent the SD. Inhibition is expressed as relative suppression in reference to the proliferation of T cells transduced with the empty vector.

Previous studies indicate that transduction of FOXP3fl and FOXP3Δ2 is sufficient to render human CD4+CD25− cells into Treg-like suppressor cells [8], [15]. To investigate whether also FOXP3Δ2Δ7 confers suppression we transduced freshly isolated CD4+CD25− T cells with the three human FOXP3 isoforms. Gene transfer was performed first with CD25− mouse T cells to prevent interference with endogenous FOXP3 expression known to by induced in human T cells upon activation [11], [25]. The effect of human FOXP3 was compared to murine Foxp3 or the ‘empty’ retroviral control vector. Suppression by transduced cells was determined by FACS after co-culture with CFSE-labelled CD4+CD25− responder cells. As expected, proliferation of responder cells was completely blocked in the presence of CD25+ Treg cells freshly isolated from mice or with cells transduced with mouse Foxp3 (Fig. 5*A*). Suppression was also observed with mouse cells transduced with human FOXP3fl and FOXP3Δ2. In contrast, proliferation of responder cells in the presence of FOXP3Δ2Δ7 transduced cells was completely unaffected. The discrepancy was particularly evident when titrating the FOXP3-transduced T cells, suggesting that FOXP3Δ2Δ7 apparently fails to transfer any detectable suppressor function (Fig. 5*B*).

### FOXP3Δ2Δ7 fails to induce Treg-specific phenotype

**Figure 6:**
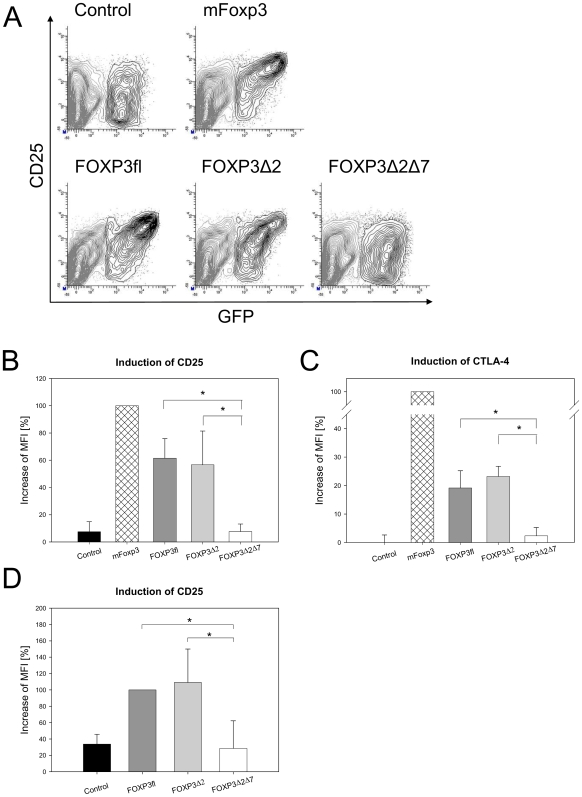
Absence of exon 7 prevents the induction of Treg associated surface markers. (A) Mouse CD4+CD25− T cells were transduced with retroviral vectors encoding for FOXP3-IRES-GFP. An increase of CD25 expression in GFP+ cells was observed in T cells transduced with murine Foxp3 (mFoxp3), human FOXP3fl and FOXP3Δ2, but not with empty vector (Control) or with FOXP3Δ2Δ7. Data shown is representative for six independent experiments. (B) Induction of CD25 expression was quantified by the relative increase of the mean fluorescence intensity (MFI) from GFP^low^ to GFP^high^ transduced cells. The increase is shown in reference to the increase by mFoxp3. Data represent the mean of six individual experiments. Error bars represent SD; significance was determined by using the unpaired student's t-test. (C) Induction of CTLA-4 expression. Relative expression was determined as described above. Data represent the mean of three individual experiments. Error bars represent SD; significance was determined by using the unpaired student's t-test. (D) Transduction of human CD4+CD25− T cells. The relative increase in expression is shown for CD25. The experiment was carried out as described above except that the relative increase in shown in reference to human FOXP3fl. Data represent the mean of four individual experiments. Error bars represent SD; significance was determined by using the unpaired student's t-test.

Since FOXP3Δ2Δ7 is apparently not able to transfer suppressive capacity, we investigated whether at least surface markers associated with Treg cells were induced. For this, mouse CD4+CD25− T cells were transduced with retroviral FOXP3 constructs in which their expression was linked to GFP (FOXP3-IRES-GFP). After 4 days post-transduction the cells were analysed by FACS for GFP-, CD25− and CTLA-4-expression. Transduction with FOXP3fl and FOXP3Δ2 as well as murine Foxp3 induced the expression of CD25. The increase was proportional to the amount of Foxp3, indicated by the GFP fluorescence (Fig. 6*A*). In contrast, cells transduced with FOXP3Δ2Δ7 or control vector did not show any upregulation. The quantification of the induction revealed that both human FOXP3fl and FOXP3Δ2 induced CD25 expression to a level of approximately 60% compared to murine Foxp3, while no enhancement was observed with FOXP3Δ2Δ7 (Fig. 6*B*). Similar results were also observed with CTLA-4 (Fig. 6*C*). Compared to murine Foxp3, transduction with human FOXP3fl and FOXP3Δ2 provoked an increase of CTLA-4 expression to 40 and 30%, respectively, FOXP3Δ2Δ7 showed no induction of CTLA-4.

The same effect was observed also when human CD4+CD25− T cells were transduced with the construct. In line with previous reports [8], [15], the overexpression of both FOXP3fl and FOXP3Δ2 enhanced the expression of CD25 (Fig. 6*D*). In contrast, FOXP3Δ2Δ7 failed to induce any significant increase of the marker although western blot analysis confirmed the expression of the transduced FOXP3 isoform in addition to lower amounts of activation-induced endogenous FOXP3fl and FOXP3Δ2 (data not shown).

### FOXP3Δ2Δ7 exhibits a dominant negative effect on FOXP3-induced CD25 expression

**Figure 7:**
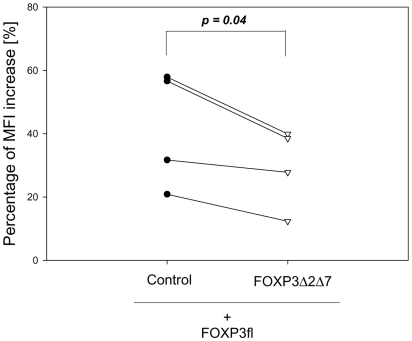
FOXP3Δ2Δ7 can act as dominant negative inhibitor of FOXP3fl. Mouse CD4+CD25− T cells were transduced with retroviral vector pairs encoding for FOXP3fl-IRES-CFP / IRES-GFP or FOXP3fl-IRES-CFP / FOXP3Δ2Δ7-IRES-GFP. The relative increase in CD25 expression was measured by determining the increase in mean fluorescence intensity (MFI) from GFP+ single transduced cells to GFP+CFP+ double transduced cells. The FOXP3fl-induced CD25 expression was significantly lower in the presence of FOXP3Δ2Δ7. Data represent the mean of four individual experiments; p-value was determined by using the paired student's t-test.

Despite the lack of function, FOXP3Δ2Δ7 was able to recruit transcription factors such as NF-κB, NFAT or RUNX1 (see Fig. 4). By competing for these factors with the other two isoforms, it may therefore act as dominant negative inhibitor. To address this question, murine CD4+CD25− T cells were co-transduced with FOXP3fl and FOXP3Δ2Δ7. To identify cells expressing both isoforms retroviral IRES-constructs were used that encoded for two different fluorescent proteins (FOXP3fl-IRES-CFP, FOXP3Δ2Δ7-IRES-GFP or the ‘empty’ IRES-GFP control vector). When comparing FOXP3fl-IRES-CFP/IRES-GFP transduced cells with FOXP3fl-IRES-CFP/FOXP3Δ2Δ7-IRES-GFP transduced cells, we observed indeed a significant inhibition of the FOXP3-induced CD25 expression (Fig. 7). In all cases, a reduction in the increase of CD25 related MFI was observed. Thus, at least in principle FOXP3Δ2Δ7 can exhibit a dominant negative effect on the functionally active full-length form of human FOXP3.

## Discussion

In this study we showed that in addition to the two dominant isoforms FOXP3fl and FOXP3Δ2 a third splice variant, FOXP3Δ2Δ7, exists, which still binds to transcription factors, such as NFAT, NF-kB and RUNX1. While it lacks apparently its suppressive functions, it is able to inhibit FOXP3fl in a dominant negative manner. The effect is caused by the loss of exon 7, encoding for a typical leucine zipper domain usually used for homo-/heterodimerization.

Alternative splicing is a mechanism commonly used by eukaryotes to increase the functional variety of proteins by exon creation and/or loss [26]. It is estimated that about 40–60% of human genes are alternatively spliced. In the case of FOXP3 the loss of exon 2 eliminates some of its repressor function, as it abrogates the interaction with RORγt, the transcription factor driving the differentiation towards Th17 [14]. In addition to this, FOXP3Δ2Δ7 also lacks the leucine zipper domain in exon 7, which has been thought to be particularly crucial for the homodimerization of FOXP3. The latter assumption was based on the impaired dimerization reported for IPEX mutations ΔK250 and ΔE251 targeting this region [19], [20]. Surprisingly, however, the complete loss of this region in FOXP3Δ2Δ7 did clearly not impair homodimerization with itself or any of the other FOXP3 isoforms. Also the homologue region in the N-myc protein was initially thought to be required for homodimerization [27]. Recent findings showed instead that the N-myc leucine zipper mediates exclusively heterodimerization with other binding partners [28], [29]. In analogy, also exon 7 may therefore primarily mediate interactions with leucine zipper proteins other than FOXP3.

In contrast to the IPEX-linked single amino acid deletions ΔK250 and ΔE251 [19], [20], the complete absence of exon 7 did not affect NFAT-mediated transcriptional repression. In this respect, the single amino acid deletions seem to have a more dramatic effect on the overall structure of the protein than the loss of the domain. Moreover, also the interaction with NF-kB was apparently not disturbed by the missing exon. This, however, may depend on the experimental system, since others have reported an isoform-specific effect in transfected Jurkat cells [30]. Recent data obtained by crystal structure analysis and co-immunoprecipitation experiments mapped the binding site of the NFAT RelA domain to the forkhead domain of FOXP3 [10], whereas RUNX1 targets a region starting at the c-terminus of exon 7 [21]. The phenotype induced by a FOXP3 mutant failing to bind RUNX1 is characterized by a loss of suppression and abrogation of CD25 induction [21]. Although this would have matched the phenotype induced by FOXP3Δ2Δ7, co-immunoprecipitation experiments clearly showed that RUNX1 is still able to bind.

Smith et al. reported that FOXP3Δ2Δ7 inhibits IL-2 release and CD69 upregulation in T cells cotransfected with a chimeric CD28/TCRζ receptor [16]. In our system, however, FOXP3 transduction had no influence on CD69 expression (data not shown). In contrast to FOXP3fl and FOXP3Δ2, FOXP3Δ2Δ7 does not increase CD25 and CTLA-4 expression. Moreover, FOXP3Δ2Δ7 expressing T cells are not able to inhibit lymphoproliferation. Thus, while FOXP3Δ2Δ7 is able to recruit transcription factors, it is apparently functionally inactive with regard to the suppressor functions commonly linked to FOXP3. Notably, co-expression of FOXP3Δ2Δ7 and FOXP3fl resulted in a reduction of FOXP3-induced CD25 expression but no FOXP3Δ2Δ7-mediated downregulation of CD25 was observed after exogenous overexpression in freshly isolated CD4+CD25+ human Treg cells (data not shown). Although FOXP3Δ2Δ7 clearly exhibits a dominant negative effect, at this point it is not clear yet if it is caused solely by the competition for transcription factors or also by the occupation of DNA binding sites. Also still unclear is whether FOXP3Δ2Δ7 lost any ability to induce a Treg-like expression pattern or if some direct inducer/repressor function is conserved.

The crucial question relates to the physiological role of the FOXP3Δ2Δ7 splice variant. In natural Treg, FOXP3Δ2Δ7 is present at rather low copy numbers. The dominant negative effect observed in vitro after ectopic expression is therefore unlikely to have any significant impact in these cells. Others have shown, however, that FOXP3Δ2Δ7 is overexpressed by certain malignant cells. Sézary Syndrome is an aggressive variant of cutaneous T cell lymphoma. A recent study suggests that in most cases the transformed T cells express FOXP3, but only FOXP3Δ2 and, even more abundantly, FOXP3Δ2Δ7 [30]. Moreover, FOXP3 also plays a role in breast cancer, as FOXP3fl acts as repressor of Her-2 [31]. Future studies have to establish, however, whether the isoform actually plays a role in cancer pathogenesis.

## Materials and Methods

### Cell purification

Balb/c mice purchased from Charles River Laboratories were housed in the animal facility of the Max Delbrück Center for Molecular Medicine and handled in accordance with the institutional guidelines. Lymphocyte populations were isolated from spleen and lymph node by using ‘mouse CD4+CD25+ Regulatory T cell Isolation Kit’ (Miltenyi) according to manufacturer's instructions. Human PBMCs from healthy volunteers were isolated by Ficoll centrifugation (GE Healthcare); Treg cells were isolated using ‘human CD4+CD25+ Regulatory T cell Isolation Kit’ (Miltenyi) according to manufacturer's instructions. Purity of magnetic bead isolated cell populations was always greater than 92%. All animal experiments were approved by the Landesamt für Arbeitsschutz, Gesundheitsschutz und Technische Sicherheit (Berlin, Germany). Approval for use of human PBMCs was obtained from the MDC institutional review boards for these studies. Informed consent was obtained in accordance with the Declaration of Helsinki.

### Cell culture

T cells were cultured in RPMI/10% FCS, supplemented with, 2 mM L-Glutamine, 1 mM non-essential amino acids, 10 mM HEPES, 50 µM β-Mercaptoethanol (all from Invitrogen), 100 IU/ml Penicillin/100 µg/ml Streptomycin (Cambrex) and 1 mM Pyruvate (Roth). HEK293T cells and NIH3T3 cells were cultured in DMEM/5% FCS medium, supplemented with 2 mM L-Glutamine, 1 mM non-essential amino acids, 50 µM β-Mercaptoethanol (all from Invitrogen) and 100 IU/ml Penicillin/100 µg/ml Streptomycin (Cambrex). Retroviral packaging cells, Platinum E (PlatE) cells [32], were cultured in DMEM/5% FCS and selected packaging-competent with Puromycin (1 µg/ml) and Blasticidin (10 µg/ml) and cultured at 32°C to maximize ecotropic virus titer.

### *RT-PCR*

mRNA was isolated with Trizol (Invitrogen), cDNA derived from human Treg cells was generated by using poly-dT primer and RT-Superscript II (Invitrogen). FOXP3 isoforms were obtained from this cDNA using the primer pair: 5′-ACGCTACTCGAGGCCACCATGCCCAACCCCAGGCCTG-3′ (sense) and 5′- TGCGAGGAATTCTCAGGGGCCAGGTGTAGGG-3′ (antisense). RT-PCR was carried out with exon overlapping primer pairs: 5′-CAGCTGCAGCTGCCCACACTG-3′ (sense) and 5′-GCCTTGAGGGAGAAGACC-3′ (antisense) for FOXP3fl, 5′-CAGCTGCAGCTCTCAACGGTG-3′ (sense) and 5′-GCCTTGAGGGAGAAGACC-3′ (antisense) for FOXP3Δ2 and 5′-GAGCAGCAGGCATCATCCG-3′ (sense) and 5′-CTGGGAATGTGCTGTTTCC-3′ (antisense) for FOXP3Δ2Δ7. RT-PCR reactions were performed using RealMasterMix (Eppendorf) according to the manual. Cycling conditions were 2 min at 95°C, followed by 55 cycles of 15 sec 95°C, 45 sec 58°C and 30 sec 68°C, finally 1 min 95°C and 1 min 55°C; at the end of the PCR specific amplification products were confirmed by melting curves and 2% agarose gel electrophoresis. FOXP3 expression was normalized to Hypoxanthin-Guanin-Phosphoribosyltransferase (HPRT) and GAPDH expression.

### Constructs

FOXP3 isoforms were cloned into pcDNA3.1+ (Invitrogen) for reporter gene assays, into pCMV-Tag2 (provided by S. Sakaguchi, Kyoto University, Japan) for FLAG-tagged FOXP3 co-immunoprecipitation experiments, and into retroviral MIGR1 vector (provided by S. Sakaguchi, Kyoto University, Japan) containing an internal ribosome entry site (IRES)-GFP. Replacement of GFP with cyan fluorescence protein (CFP) into MIGR1 was performed via NcoI/SalI cloning, using 5′-ACGCTCCATGGGCCACCATGGTGAGCAAGGGCGAG-3′ (sense) and 5′-TGCGAGTCGACCTACTTGTACAGCTCGTCCATG-3′ (antisense) and pLP-ECFP-C1 (Clontech) as template. FLAG-tagged RUNX1 co-immunoprecipitation experiments were carried out with pCMV-RUNX1-Tag2 (provided by S. Sakaguchi, Kyoto University, Japan). For translocation experiments FOXP3 isoforms and FOXP3ΔFKH (lacking C-terminal 104aa) were cloned into pcDNA3.1+, followed by N-terminal fusion to GFP, using 5′-ACGCTGGGATCCGCCGCCACCATGGCCACAACCATGGTGAGC-3′ (sense) and 5′-TGCGACGAATTCCTTGTACAGCTCGTCCATGCC-3′ (antisense). Integrity of constructs was confirmed by sequencing.

### Transfection and virus production

HEK293T cells (ATCC) were transfected by using Fugene transfection reagent (Roche). Ecotropic virus was produced by transfection of PlatE cells with MIGR1 constructs as described [5]. Amphotropic virus was produced in HEK293T cells after transfection with MIGR1 constructs, pALF-10A1 and pcDNA3.1MLVg/p as described [33].

### Transduction

Retroviral gene transfer of primary murine CD4+ T cells were performed according to previous reports [5]. Briefly, 2×10^6^ freshly isolated MACS-isolated murine CD4+CD25− cells were incubated with 7 µg/ml plate bound α-CD3 (145.2C11, purified at the MDC from cell supernatant), 1×10^6^ irradiated APCs and 100 IU/ml IL-2 (Roche Diagnostics) in a 24-well plate over night. Spin-infection was carried out on the following 2 days with filtered virus supernatant, in the presence of 7 µg/ml Polybrene at 1800 rpm, 90 min, 32°C. 10 IU/ml IL-2 was supplemented on day 2 after cell isolation. For transduction of human cells 2×10^6^ freshly isolated human CD4+CD25− cells were incubated with 5 µg/ml plate bound α-CD3 (OKT3, eBioscience) and 1 µg/ml α-CD28 (28.2, BD Biosciences) and 100 IU/ml IL-2 in a 24-well plate for 36 h. Spin infection was carried out as described [33]. Gene expression patterns were measured at day 6 by flow cytometry.

### Flow cytometry

Gene expression was quantified by antibody staining of mCD4 (GK1.5), mCD25 (PC61), hCD4 (RPA-T4) and hCD25 (M-A251) (all antibodies from BD Biosciences). For intracellular α-CTLA-4 staining (UC10-4F10–11, BD Biosciences), cells were treated 6 h with PMA (50 ng/ml), Ionomycin (1 mM) and Brefeldin A (2 µg/ml, Sigma-Aldrich). Dead cells were excluded from analysis by life gating using propidium iodide. FACS analysis was carried out on a LSR II (BD Biosciences), cells were sorted on a FACSAria (BD Biosciences). Data were analysed using FACSDiva software (BD Biosciences). Induction of gene expression in FOXP3-IRES-GFP transduced cells was performed by comparing MFI values of GFP^low^ and GFP^high^ expressing cells. In GFP/CFP double transduction experiments to determine the dominant negative effect of FOXP3Δ2Δ7, the increase in CD25 expression was compared in response to IRES-GFP / FOXP3fl-IRES-CFP and FOXP3Δ2Δ7-IRES-GFP / FOXP3fl-IRES-CFP.

### Suppression assay

Freshly isolated murine CD4+CD25− cells were labelled with 2,5 µM CFSE (Invitrogen) and plated at 1×10^5^/well in 96-well plates, stimulated with 3 µg/ml plate bound α-CD3 (145.2C11, purified at the MDC) and 5×10^4^ CD4- cells as APC, irradiated at 30 Gy. Responder cells were co-cultured with FOXP3-transduced CD4+ cells, sorted before based on the corresponding GFP or CFP fluorescence, or with freshly isolated CD4+CD25+ regulatory T cells. Proliferation was determined after 4 days by flow cytometry by recording CFSE dilutions.

### Reporter gene assay

Luciferase reporter assays were carried out with the following constructs: pNFAT-TA-Luc (Clontech) and pNF-κB-Luc (Stratagene) (both provided by R. Baumgras, DRFZ, Germany). Endotoxin was removed with TritonX-114 [34]. Endotoxin levels were measured with limulus amebocyte lysate assay (QCL-1000, Cambrex) according to manufacturer's instructions. NIH3T3 cells were transiently transfected with 1 µg total DNA using Lipofectamine 2000 (Invitrogen). The ratio of transfected plasmids was: 1 part pRL-CMV (Promega), 10 parts indicated firefly luciferase reporter gene construct and 50 parts indicated FOXP3 expression construct. Cells were lysed after 48 h; samples were measured as triplicates; variations in the transfection rate were compensated by normalizing to the Renilla luciferase activity.

### Western blotting and Immunoprecipitation

Protein interactions were detected by co-immunoprecipitation as previously described [21]. Briefly, HEK293T cells were transfected with pcDNA3.1+ constructs encoding for Flag-tagged and non-tagged proteins. After 3 days cells were lysed in non-denaturating buffer (150 mM NaCl; 50 mM Tris-HCl, pH: 7,5; 1% Nonidet P-40; 0,5% sodium deoxycholate) containing protease inhibitor (Roche). Lysates were centrifuged (5 min, 13000 rpm) and the supernatant was incubated 2 h at 4°C with α-FLAG M2 affinity gel (SigmaAldrich) while gently rocking. The beads were centrifuged (30 sec, 1000 rpm) and washed 6 times with cold lysis buffer. Samples were denatured by 95°C for 10 min and separated on 10% SDS-PAGE. Proteins were transferred to Immobilon P membrane (Millipore) using Transblot SD (Biorad) at 0,8 mA/cm^2^ for 1 h. Membranes were blocked with 2,5% milk, and incubated with isoform-non specific α-FOXP3 (e7979, eBioscience), followed by washing and incubation with secondary HRP-conjugated antibody (Dako). Protein bands were visualized by ‘ECL Plus Western Blotting Detection Reagents’ (GE Healthcare).

### Statistics

Statistical analysis was performed with 2-tailed paired or unpaired student's t-test using Sigmaplot software (San Jose). P-values less than 0,05 were considered statistically significant. All error bars represent the standard deviation (SD).

## References

[1] Sakaguchi S, Sakaguchi N, Asano M, Itoh M, Toda M (1995). Immunologic self-tolerance maintained by activated T cells expressing IL-2 receptor alpha-chains (CD25). Breakdown of a single mechanism of self-tolerance causes various autoimmune diseases.. J Immunol.

[2] Takahashi T, Tagami T, Yamazaki S, Uede T, Shimizu J (2000). Immunologic self-tolerance maintained by CD25(+)CD4(+) regulatory T cells constitutively expressing cytotoxic T lymphocyte-associated antigen 4.. J Exp Med.

[3] Sakaguchi S, Yamaguchi T, Nomura T, Ono M (2008). Regulatory T cells and immune tolerance.. Cell.

[4] Borsellino G, Kleinewietfeld M, Di Mitri D, Sternjak A, Diamantini A (2007). Expression of ectonucleotidase CD39 by Foxp3+ Treg cells: hydrolysis of extracellular ATP and immune suppression.. Blood.

[5] Hori S, Nomura T, Sakaguchi S (2003). Control of regulatory T cell development by the transcription factor Foxp3.. Science.

[6] Brunkow ME, Jeffery EW, Hjerrild KA, Paeper B, Clark LB (2001). Disruption of a new forkhead/winged-helix protein, scurfin, results in the fatal lymphoproliferative disorder of the scurfy mouse.. Nat Genet.

[7] Bennett CL, Christie J, Ramsdell F, Brunkow ME, Ferguson PJ (2001). The immune dysregulation, polyendocrinopathy, enteropathy, X-linked syndrome (IPEX) is caused by mutations of FOXP3.. Nat Genet.

[8] Yagi H, Nomura T, Nakamura K, Yamazaki S, Kitawaki T (2004). Crucial role of FOXP3 in the development and function of human CD25+CD4+ regulatory T cells.. Int Immunol.

[9] Bettelli E, Dastrange M, Oukka M (2005). Foxp3 interacts with nuclear factor of activated T cells and NF-kappa B to repress cytokine gene expression and effector functions of T helper cells.. Proc Natl Acad Sci U S A.

[10] Wu Y, Borde M, Heissmeyer V, Feuerer M, Lapan AD (2006). FOXP3 controls regulatory T cell function through cooperation with NFAT.. Cell.

[11] Allan SE, Passerini L, Bacchetta R, Crellin N, Dai M (2005). The role of 2 FOXP3 isoforms in the generation of human CD4+ Tregs.. J Clin Invest.

[12] Du J, Huang C, Zhou B, Ziegler SF (2008). Isoform-specific inhibition of ROR alpha-mediated transcriptional activation by human FOXP3.. J Immunol.

[13] Ichiyama K, Yoshida H, Wakabayashi Y, Chinen T, Saeki K (2008). Foxp3 inhibits RORgammat-mediated IL-17A mRNA transcription through direct interaction with RORgammat.. J Biol Chem.

[14] Zhou L, Lopes JE, Chong MM, Ivanov II, Min R (2008). TGF-beta-induced Foxp3 inhibits T(H)17 cell differentiation by antagonizing RORgammat function.. Nature.

[15] Aarts-Riemens T, Emmelot ME, Verdonck LF, Mutis T (2008). Forced overexpression of either of the two common human Foxp3 isoforms can induce regulatory T cells from CD4(+)CD25(−) cells.. Eur J Immunol.

[16] Smith EL, Finney HM, Nesbitt AM, Ramsdell F, Robinson MK (2006). Splice variants of human FOXP3 are functional inhibitors of human CD4+ T-cell activation.. Immunology.

[17] Ziegler SF (2006). FOXP3: of mice and men.. Annu Rev Immunol.

[18] Landschulz WH, Johnson PF, McKnight SL (1988). The leucine zipper: a hypothetical structure common to a new class of DNA binding proteins.. Science.

[19] Chae WJ, Henegariu O, Lee SK, Bothwell AL (2006). The mutant leucine-zipper domain impairs both dimerization and suppressive function of Foxp3 in T cells.. Proc Natl Acad Sci U S A.

[20] Li B, Samanta A, Song X, Iacono KT, Brennan P (2007). FOXP3 is a homo-oligomer and a component of a supramolecular regulatory complex disabled in the human XLAAD/IPEX autoimmune disease.. Int Immunol.

[21] Ono M, Yaguchi H, Ohkura N, Kitabayashi I, Nagamura Y (2007). Foxp3 controls regulatory T-cell function by interacting with AML1/Runx1.. Nature.

[22] Schubert LA, Jeffery E, Zhang Y, Ramsdell F, Ziegler SF (2001). Scurfin (FOXP3) acts as a repressor of transcription and regulates T cell activation.. J Biol Chem.

[23] Lopes JE, Torgerson TR, Schubert LA, Anover SD, Ocheltree EL (2006). Analysis of FOXP3 reveals multiple domains required for its function as a transcriptional repressor.. J Immunol.

[24] Hermiston ML, Xu Z, Weiss A (2003). CD45: a critical regulator of signaling thresholds in immune cells.. Annu Rev Immunol.

[25] Allan SE, Crome SQ, Crellin NK, Passerini L, Steiner TS (2007). Activation-induced FOXP3 in human T effector cells does not suppress proliferation or cytokine production.. Int Immunol.

[26] Modrek B, Lee CJ (2003). Alternative splicing in the human, mouse and rat genomes is associated with an increased frequency of exon creation and/or loss.. Nat Genet.

[27] Chatila TA, Blaeser F, Ho N, Lederman HM, Voulgaropoulos C (2000). JM2, encoding a fork head-related protein, is mutated in X-linked autoimmunity-allergic disregulation syndrome.. J Clin Invest.

[28] Nair SK, Burley SK (2003). X-ray structures of Myc-Max and Mad-Max recognizing DNA. Molecular bases of regulation by proto-oncogenic transcription factors.. Cell.

[29] Lebel R, McDuff FO, Lavigne P, Grandbois M (2007). Direct visualization of the binding of c-Myc/Max heterodimeric b-HLH-LZ to E-box sequences on the hTERT promoter.. Biochemistry.

[30] Krejsgaard T, Gjerdrum LM, Ralfkiaer E, Lauenborg B, Eriksen KW (2008). Malignant Tregs express low molecular splice forms of FOXP3 in Sezary syndrome.. Leukemia.

[31] Zuo T, Wang L, Morrison C, Chang X, Zhang H (2007). FOXP3 is an X-linked breast cancer suppressor gene and an important repressor of the HER-2/ErbB2 oncogene.. Cell.

[32] Morita S, Kojima T, Kitamura T (2000). Plat-E: an efficient and stable system for transient packaging of retroviruses.. Gene Ther.

[33] Leisegang M, Engels B, Meyerhuber P, Kieback E, Sommermeyer D (2008). Enhanced functionality of T cell receptor-redirected T cells is defined by the transgene cassette.. J Mol Med.

[34] Petsch D, Anspach FB (2000). Endotoxin removal from protein solutions.. J Biotechnol.

